# From Small Peptides to Large Proteins against Alzheimer’sDisease

**DOI:** 10.3390/biom12101344

**Published:** 2022-09-22

**Authors:** Pasquale Picone, Tiziana Sanfilippo, Sonya Vasto, Sara Baldassano, Rossella Guggino, Domenico Nuzzo, Donatella Bulone, Pier Luigi San Biagio, Emanuela Muscolino, Roberto Monastero, Clelia Dispenza, Daniela Giacomazza

**Affiliations:** 1 Istituto per la Ricerca e l’Innovazione Biomedica, Consiglio Nazionale delle Ricerche, Via U. La Malfa 153, 90146 Palermo, Italy; 2 Dipartmento of Scienze Biologiche, Chimiche, Farmaceutiche e Tecnologiche (STEBICEF), University of Palermo, 90128 Palermo, Italy; 3 Ambulatorio di Nutrizione Clinica ASP Palermo, Via G. Cusmano 24, 90141 Palermo, Italy; 4 Anestesia e Rianimazione, Presidio Ospedaliero “S. Cimino”, 90141 Termini Imerese, Italy; 5 Istituti Euro-Mediterranei di Scienza e Tecnologia (IEMEST), Via M. Miraglia 20, 90139 Palermo, Italy; 6 Istituto di Biofisica, Consiglio Nazionale delle Ricerche, Via U. La Malfa 153, 90146 Palermo, Italy; 7 Dipartimento di Ingegneria, Università degli Studi di Palermo, Viale delle Scienze, Bldg 6, 90128 Palermo, Italy; 8 Dipartimento di Biomedicina, Neuroscienze e Diagnostica Avanzata, Università degli Studi di Palermo, Via del Vespro 129, 90127 Palermo, Italy

**Keywords:** Alzheimer’s disease, Tau protein, neurofibrillary tangles, amyloid-beta protein: amyloid fibrillation

## Abstract

Alzheimer’s disease (AD) is the most common neurodegenerative disorder in the elderly. The two cardinal neuropathological hallmarks of AD are the senile plaques, which are extracellular deposits mainly constituted by beta-amyloids, and neurofibrillary tangles formed by abnormally phosphorylated Tau (*p*-Tau) located in the cytoplasm of neurons. Although the research has made relevant progress in the management of the disease, the treatment is still lacking. Only symptomatic medications exist for the disease, and, in the meantime, laboratories worldwide are investigating disease-modifying treatments for AD. In the present review, results centered on the use of peptides of different sizes involved in AD are presented.

## 1. Introduction

Alzheimer’s disease is a complex progressive neurodegenerative disorder affecting older people and strongly interfering with the daily activities of patients [1]. Although it is now clear that the disease starts at least ten years before its clinical manifestation, symptoms affect memory, language, orientation, and judgement and, gradually, the disease proceeds toward a complete cognitive and functional decline [2].

In particular, the disease is preceded by a preclinical stage, the so-called subjective cognitive decline (SCD) [3], in which the subjects self-experience persistent deterioration in cognitive functioning in comparison with a prior normal status, but cognitive performance and functional abilities are not impaired. Subsequently, the prodromal phase of the disease appears, the so-called mild cognitive impairment (MCI) [4], which is characterized by impaired cognitive performance with none or only slight fault of patients’ functional abilities. Finally, the full spectrum of AD emerges with a multidomain impairment involving cognitive, behavioral, and motor functions with inherent problems of disability and reduced patient quality of life [5]. Overall, the clinical stages of AD are associated with specific biomarker progression and neuropathology. Concerning biomarkers, the Amyloid/Tau/Neurodegeneration (ATN) framework has been proposed to highlight the biological state of the disease [6]. This classification scheme has revealed a clinically relevant prognostic value for the evolution of cognitive decline in clinical practice [7].

Regarding neuropathology, Braak staging suggests that Tau pathology starts in the entorhinal cortex and progressively affects other brain regions [8]. Stages I-II concern the preclinical phase of AD and affect the transenthorinal region of the brain; in the stages III-IV, in which the limbic area is interested, the first clinical signs of the disease appear, thus characterizing MCI and mild AD; the stages V-VI regard the fully blown disease, now extended in the isocortical areas.

## 2. The Hallmark Lesions of AD: β-Amyloid and Tau Proteins

AD is characterized by neuron loss and increasing accumulation of neurofibrillary tangles formed by Tau protein inside the cells and the presence of amyloid plaques, mainly constituted by extracellularly aggregated amyloid beta-protein [9].

### 2.1. Amyloid β-Peptide (Abeta)

Amyloid-β peptide (Abeta), ranging from 37 to 43 residues with different aggregation propensity, is obtained by the enzymatic cleavage of the Amyloid Precursor Protein (APP), a large transmembrane metal binding protein of 695–770 aminoacids [10,11].

The physiological role of APP is not yet fully understood. There are indications that the protein is involved in neurogenesis [12], neurite growth and long-term potentiation by regulation of calcium release [13]. It has been demonstrated that very small concentrations (picomolars) of Abeta improves memory in mice; while, on the contrary, high Abeta levels inhibit it [14]. Antimicrobial activity, inhibition of oncogenic viruses, enhanced activation of acetylcholine and nicotinic acetylcholine receptors have been observed as physiological effects of Abeta [15].

**Figure 1:**
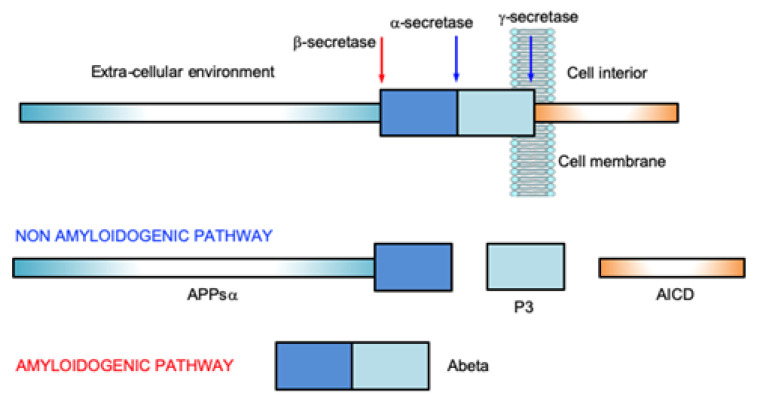
Results of the different cleavage sites of APP by the secretase enzymes.

The cleavage of APP is the result of the activity of the enzymes of the secretase family, α- (ADAM), β- (BACE1) and γ- (or Presenilins) secretases, whose sequential intervention results in the onset of the amyloidogenic or non-amyloidogenic pathway (Figure 1).

The non-amyloidogenic pathway is the consequence of the involvement of α- and γ-secretases, and it results in the formation of the APPsα, P3 and AICD (APP Intracellular Domain) fragments. While APPsα and P3 still have unknown functions, the latter moves to the nucleus and there regulates gene expressions and the apoptosis process [16]. In the amyloidogenic pathway, β- and γ-secretases operate the enzymatic cut, thus originating the Abeta fragments [17]. Recent evidence has suggested that Abeta42 can also be generated by the action of meprin β, a metalloprotease that acts at the same cleavage site of BACE1 [18].

α-Secretase activity is ascribed to the ADAM metalloproteases, among which it has been shown that ADAM 9, 10, 17 and 19 hold α-secretase action [19]. In particular, the overexpression of ADAM 10 in AD mouse reduced plaque formation and cognitive failure [20]. Due to the abundance of components of the family, it is liable that other ADAM enzymes intervene in the α-secretase complex [19].

BACE1 is present in all tissues and organs, but it reaches very high concentration levels in the brain and pancreas. Because APP is also highly expressed in the brain, the simultaneous presence of BACE 1 and APP can explain the reason why AD is a brain disease [17]. Based on these discoveries, the intuitive approach of inhibiting the activity of BACE1 had ambiguous results. If BACE1 homozygote knockout mice exhibited a complete absence of Abeta production without any physiological deficit [21], clinical trials based on BACE inhibitors did not have the same success [22]. The difficulty to selectively inhibit BACE1 without affecting the action of the other proteases in the body and to overtake the blood-brain barrier are some of the obstacles to be overcame to develop a BACE1 inhibitor therapy [23].

γ-Secretase is an enzymatic complex with auto-catalytic properties which is formed by four proteins: presenilin (PS1), nicastrin (Nct), presenilin enhancer 2 (Pen2) and anterior pharynx-defective 1 (Aph-1). Their assembly occurs in sequential steps: the first complex is formed by Nct and Aph-1, and is followed by the PS link. The last step is the binding of Pen2, which allows the auto-cleavage of PS, thus generating the N-and C-termini of the protein [24].

After the discovery that Abeta42 is the main component of the amyloid senile plaques found in brain parenchyma of AD patients, in 1992, Hardy and Higgins postulated that *“…deposition of amyloid β protein (AβP), … is the causative agent of Alzheimer’s pathology and that the neurofibrillary tangles, cell loss, vascular damage, and dementia follow as a direct result of this deposition*” [25]. This hypothesis was further corroborated by the observations that mutations in APP and presenilin genes, leading to aggregation prone Abeta fragments, are directly involved in the aggressive familial AD onset [26].

**Figure 2:**
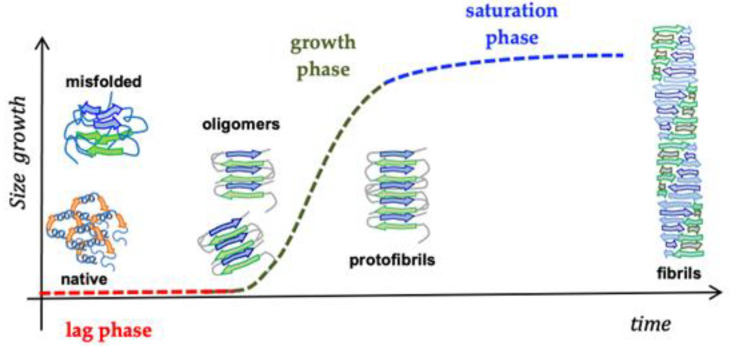
Scheme of the amyloid aggregation phases.

In vitro, the fibrillation profile of Abeta42 can be described by a three-stage process starting from the native structure of the peptide, then involving the formation of aggregation-prone intermediate species, up to the formation of mature fibrils (Figure 2) [27]. The presence of seeds, small proteinaceous aggregates, reducing the lag phase, intensely modify the aggregation kinetics, thus in vitro appropriate treatment to start from a free-aggregate sample is required [28].

A well-known polypeptide sharing many biophysical and physiological features with Abeta, and able to interact with it, is the islet amyloid polypeptide hormone, known as IAPP or amylin, identified for the first time in 1987 [29]. IAPP is secreted by the beta-cells of the pancreatic islets of Langerhans, which also secrete insulin, and has a role in the control of the blood glucose level [30]. The presence of IAPP on the beta-cell membranes together with presence of alterations in the membranes suggest that this interaction is responsible for the cytotoxic effect of these formations [31].

The primary sequence of the peptide is extremely conserved in organisms, for example, human and mouse IAPPs differ by only six amino acids. The formation of IAPP deposits is intrinsically associated with type 2 diabetes (T2D) because more than 70% diabetic patients present this type of amyloid formation [32]. The number of aggregates appears to be correlated with the pathology severity, as evidenced by autopsies. The human IAPP (hIAPP) accounts for 37 amino acids with an S-S bridge; it is an intrinsically disordered protein, with few alpha-helical and beta-sheet components. As Abeta, IAPP is non-toxic in its monomeric form, conversely it exhibits high toxicity levels in the beta-rich amyloid aggregated structures [33]. Another similarity with Abeta is that IAPP fibrillation occurs by the formation of nuclei with a latency phase whose duration is dependent on the concentration, and proceeds with the addition of monomers or oligomers to both fibril terminations [34].

Recent studies highlighted the direct interaction of IAPP with Abeta. Each of the two proteins can mutually participate in the aggregation of the other by acting as seed and inducing the formation of heterocomplexes [35]. Abeta42-hIAPP heterocomplex mixtures showed greater ability in inducing cell death through the formation of large amorphous aggregates, although they are generally considered less toxic than soluble oligomers [36]. Abeta42-hIAPP heterocomplexes are also able to bind some cellular receptors and intervene in cellular pathways, inducing cell damage and death [36]. In SHSY-5Y model cells, a concentration-dependent effect of hIAPP was observed in promoting the uptake of Abeta42, implying that the Aβ42-hIAPP heterocomplexes have a synergistical ability in promoting amyloid structure formation in the brain [35]. The interactions of the Abeta-hIAPP heterocomplexes with membranes is important in understanding their pathological role in AD andT2D. Recent molecular dynamics (MD) simulation studies showed that Abeta-hIAPP interacts more strongly with the lipid bilayers due to electrostatic interactions and the formation of Ca^2+^ bridges [37].

### 2.2. Tau Protein

Tau is a microtubule-associated protein, mainly expressed in the axons of neurons, deputed to mantain the microtubules that ensure the structural stability of the cell and allow the organelles, vesicles and proteins to move through the cell [38,39]. Several dysfunctions of Tau have been identified, constituting the family of neurogenerative diseases known as Tauopathies, including AD [40].

**Figure 3:**
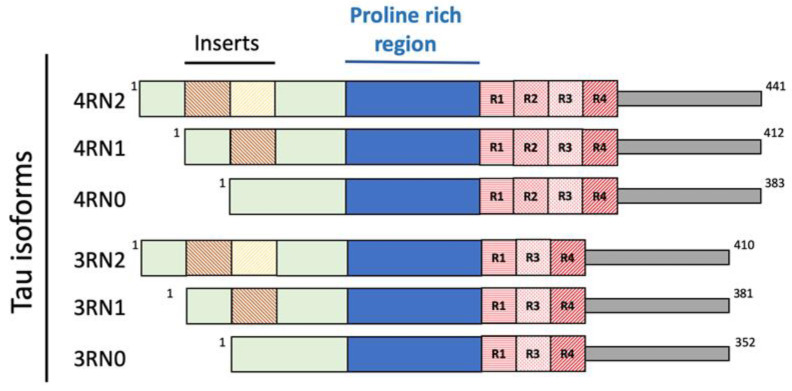
Results of the different human MAPT gene splicing in the expression of Tau isoforms.

In solution, Tau possesses a harpin-like disordered and unfolded structure [39]. Because of different splicing during human MAPT gene translation, six different isoforms of Tau are present: 3R0N (352 aminoacids, aa.), 3R1N (381 aa.), 3R2N (410 aa.), 4R0N (383 aa.), 4R1N (412 aa.) and 4R2N (441 aa.), depending on the absence (0N) or presence of one (1N) or two (2N) inserts at the N terminus of the protein (Figure 3).

In the fetal brain, the 3RN0 isoform is the only one present, while in the adult one and under physiological conditions, the ratio between the 3N and 4N isoforms is 1:1; in Tauopathies an altered 3N:4N ratio is observed [39], although the protein can undergo considerable variations in concentration in the different cerebral areas [41]. The longest isoform, 4RN2, possesses more than 80 potential sites (Ser, Thr and Tys residues) where phosphorylation can occur. This makes feasible a dynamical process of phosphorylation and de-phosphorylation, allowing a rapid regulation and maintenance of the microtubule structure [41].

In the normal brain only two or three Tau aminoacidic residues are phosphorylated in the proline-rich region. In the AD brain, for still unknown reasons, Tau is excessively hyperphosphorylated with up to nine phosphate groups [42], and this event causes a weakening of the interaction with microtubules leading to the disruption of the network and the aggregation of Tau into protease-resistant helical filaments [43,44]. The repeat domains of Tau (R1, R2, R3 and R4 in Figure 1) are responsible for the binding to microtubules, the 4R isoforms having a higher affinity than the 3R ones and constituting the core of the aggregated filaments [41].

Recently, a mechanism explaining the Tau aggregation has been proposed. It has been observed that, similarly to what happens in the formation process of membrane-less cell organelles, in physiological conditions, Tau may undergo reversible liquid-liquid phase separation (LLPS), giving origin to high viscosity gel-like transient droplets with elevated concentrations of the protein, with unknown physiological activity [45,46]. Electrostatic and hydrophobic interactions, respectively, at the C- and N- termini, and the β-sheet structures could stabilize the droplets [45]. The excess of hyperphosphorylation changes the charge distribution and the electrostatic interactions [46]. This event could favor the Tau detachment from the microtubule structure and the formation of soluble monomeric or dimeric free Tau in the cytoplasm. This, in turn, shifts the equilibrium toward the formation of droplets and facilitates Tau aggregation [45].

After production, Tau undergoes several post-translational modifications, such as glycosylation, glycation, deamidation, methylation and ubiquitylation [41]. Acetylation and de-acetylation are the last identified modifications occurring in the Lys residues of the repeating domains [47,48]. These changes are catalyzed by acetyltransferase or sirtuin 1 and histone deacetylase enzymes. Interestingly, Tau possesses an acetyltransferase activity, thus it can auto-catalyze the reaction in some Lys residues. Depending on the involved residues, the acetylation can inhibit the protein degradation or favor it, thus impeding phosphorylation and aggregation [47]. This discovery can open the door for therapeutic strategies against Tauopathies.

Thus, the data accumulated on Abeta, Tau and involved enzymatic pathways have pushed the research towards the identification of molecules that can counteract the processes leading to AD.

### 2.3. Proteins and Metal Ions in AD

From the last decade of the past century, many studies indicated that metal ion excess, particularly Ca^2+^, Al^3+^, Fe^2+^, Cu^2+^, Zn^2+^, and Pb^2+^, plays a crucial role in the onset of AD [49,50]. Although they exert a main role in the brain homeostasis, imbalanced metal levels may actively participate in the generation of free radical species triggering the oxidation of proteins, lipids and nuclei acids in the brain [51,52,53]. High levels of metal ions, such as Zn^2+^, Cu^2+^ and Fe^2+^, have been observed in the brain plaques of AD affected individuals, co-localizing with Abeta deposits and favoring its aggregation [54]. Furthermore, their higher concentration in CNS structures is counterbalanced by their reduction in different body districts [55,56].

Although it can appear contradictory, different concentrations of metal ions lead in vitro to different results for what concerns the Abeta fate. Excessive concentrations of Cu^2+^ and Zn^2+^ give rise to insoluble amorphous Abeta aggregates [54], and equimolar levels drive to amorphous aggregates that soon evolve into ordered fibrils [57]; low metal concentrations accelerate the fibril process formation compared to the kinetics obtained with Abeta alone [58]. Interestingly, the APP E2 domain possesses high affinity towards Cu (II) and Zn (II) ions [59]. Designing short peptides showing the ability to bind copper represents a promising approach for capturing poorly localized metal ions. [60,61].

Neuronal metallothionein 3 (MT3) is a protein having an important role in AD, involved in the maintenance of Zn^2+^ and Cu^2+^ brain homeostasis and ROS control [62,63]. The latter function is possible due to the presence of cysteines, which can extinguish the production of free radicals [62]. The MT3 levels are downregulated in the AD brain. It has been demonstrated that continuous brain infusion of MT3 protein in mice reduced the oxidation levels, neuronal apoptosis, pathological hippocampal changes, and cognitive impairment occurring in AD [64]. S100 family proteins control the Ca^2+^ levels and play an important role in neuronal maintenance [65].

Zinc transporter protein (ZnT3) is a protein responsible for Zn^2+^ concentration and release in the synaptic vesicles of the glutamatergic neurons in the brain [66]. It has been found that ZnT3 levels decreased with aging and, to a higher extent, in AD; consequently, not enough Zn^2+^ can be released, causing cognitive and memory impairment [67].

S100B is the most investigated protein among those belonging to the S100 family. Its action strongly depends on concentration. At nanomolar levels it promotes neuronal and neurite growth, while its micromolar concentration values favor neuronal apoptosis [68]. Its astrocytic overexpression is related with neuritic abnormalities in AD and high levels of interleukin-1, a hallmark of the brain AD plaques [68]. Mutations in Presenilin-1 have been found in AD familial form, and it results in the downregulation of Ca^2+^ channels and Ca^2+^ dependent mitochondrial proteins [51].

The Zn^2+^ and Fe^3+^ interaction with Tau protein support its hyperphosphorylation and aggregation, while Fe^2+^ can invert the process [69].

As evidenced, metals are strongly implicated in AD, and many proteins working inside or outside the cellular environment contribute to their regulation [70].

### 2.4. Peptide-Based Scaffolds to Target Cu Ions as Therapeutics

Nanostructured peptides with metal binding properties are promising therapeutic advancements in neurodegenerative diseases. These nanostructures interact with metal ions and influence the biological properties of several proteins involved in neurodegenerative diseases [71]. The brain copper imbalance plays an important role in Abeta aggregation and in AD neurotoxicity. Moreover, the Cu^2+^ ion bound to Abeta can induce ROS production. Histidine-containing peptides and proteins are excellent metal binders and are found in many natural systems. For this aim, Caballero and Collaborators studied three short peptides, HWH, HKCH and HAH, forming highly stable albumin-like complexes, with higher affinity for Cu^2+^ than for Abeta(1–40). These copper-chelating peptides were designed with the aim of reducing copper toxicity in AD. Furthermore, HWH, HKCH and HAH act as very efficient inhibitors of copper-mediated generation of ROS and prevent the copper-induced overproduction of toxic oligomers in the early stages of amyloid aggregation in the presence of Cu^2+^ ions [60].

It was observed that the Abeta peptides, truncated at positions 4 and 11, contain an amino-terminal copper and nickel-binding motif (ATCUN) (NH2-Xxx-Zzz-His) which gives them different coordination sites and higher affinity for Cu^2+^ than for Abeta. The results show that N-truncated peptides are still able to induce ROS production when Cu^+^ is present in the medium, although to a lesser extent than the unmodified one [61,72]. In addition, when used as competitor ligands the N-truncated peptides are not able to fully preclude Cu (Abeta(1–16))-induced ROS production [61,72]. Folk and Franz described a prochelator peptide that is enzymatically activated by β-secretase. Once activated, the cleavage product effectively sequesters copper from Abeta, prevents and disassembles amyloid-aggregation, and decreases the formation of copper-promoted ROS [73].

Tripeptide GGH was used to selectively chelate the Cu^2+^ in the Abeta-Cu complex in the presence of other metal ions. In addition, the inhibitory effect of GGH on ROS production and the cytotoxicity of the Abeta-Cu complex was demonstrated [74]. These studies may help to elucidate the mechanism of the production of Abeta-Cu complex toxicity, with evident positive effects in AD therapy [74].

## 3. Oxidative Stress and Its Involvement in AD Onset

Increasing evidence indicates that oxidative activity may be involved in the etiology of AD as well as other neurodegenerative pathologies and cancer. Under physiological conditions, free radicals, reactive oxygen species (ROS) and reactive nitrogen species (NOS), are normally produced in living cells; just consider, for example, the molecular species generated during the mitochondrial electron transport chain (ETC) and the Krebs cycle [75]. These unstable molecules, with unpaired electrons, initiate a series of reactions leading to the oxidation of proteins, lipids, and nucleic acids. However, in several cases and at low-to-moderate concentrations, free radicals play a physiological role [76]. ROS derived by the action of NADPH oxidase, a superoxide-oxidase enzyme, can fight the bacterial infection in the neutrophil phagosome [77]. Furthermore, ROS are physiologically involved in some cellular pathway signaling and in the regulation of the vascular tone, cell adhesion and apoptosis [76]. They also have a key role in the protection of adults and embryonic stem cells [78].

In healthy individuals, the excess production of free radical concentration is counteracted by the oxidative defense system, including glutathione, arginine, and citrulline; some chemical elements such as selenium and zinc; the vitamins A, C and E; the enzymes superoxide dismutase, catalase, glutathione reductase and glutathione peroxidases [79]. Aging and age-related diseases contribute to the free radical productions [80].

In AD pathology, this system appears imbalanced. Indeed, oxidatively changed nucleic acid and protein products, as well as products of lipid peroxidation and glycoxidation, are recognized as markers of oxidative harm [81]. Several markers have been found in the afflicted lesions of patients with these disorders in *postmortem* brain tissue or pre-mortem cerebrospinal fluid, plasma, serum, and urine [82]. When reactive oxygen species, particularly hydroxyl radicals, attack DNA, they can cause strand breakage, DNA-DNA and DNA-protein cross-linking, sister chromatid exchange and translocation, and the production of at least 20 oxidized base adducts. Mutation and altered protein synthesis can result from the modification of DNA nucleotides [83]. Several investigations have found an increase in the base adducts 8-hydroxyguanine (8-OHG), 8-hydroxyadenine (8-OHA), 5-hydroxycytosine (5-OHC), and 5-hydroxyuracil, a chemical degradation product of cytosine, in late-stage AD brains. Oxidized base adducts were discovered in nuclear and mitochondrial DNA in mild cognitive impairment, the earliest detectable form of AD, signifying that oxidative damage to DNA is an early event in AD and not a secondary phenomenon [80,84].

Mitochondria were also called into question as one of main the protagonists of AD onset. Age-induced mitochondrial dysfunction could be considered as one of the first events occurring in AD pathogenesis. The mitochondrial cascade hypothesis states that dysfunction of these cellular organelles interferes with the APP production, forcing the activation of the amyloidogenic pathway, the production of Abeta oligomers, and plaques formation [85,86,87]. Moreover, the altered cellular metabolism and energy production, due to mitochondrial damage, lead sequentially to abnormal axonal trafficking and irregular Ca^++^ homeostasis, nuclear damage with epigenetic DNA modifications, and synaptic dysfunction. This vicious circle feeds itself and induces neuronal death [88].

Based on this finding, counteracting the onset of oxidative stress is important to avoid or reduce the onset of AD.

## 4. The Antioxidant Properties of Egg-Derived Peptides

Proteins are huge biomolecular and macromolecular structures made up of one or more long chains of amino acid residues. Proteins serve a wide range of roles within animals, including catalyzing metabolic reactions, providing structure to cells and organisms, DNA replication, transporting chemicals, and responding to stimuli. A polypeptide is a linear chain of amino acid residues. Short polypeptides with fewer than 20–30 residues are rarely regarded as proteins and are often referred to as peptides; this is why peptides can be created by the enzymatic digestion of proteins.

Today, peptides are viable alternatives to chemical medications. They are important regulators of biological functions with high biological activity, selectivity, and low toxicity. Unfortunately, the short half-life of peptide medicines in vivo can substantially impede their development. Peptides are generally rapidly destroyed by proteases, which poses challenges for administration and transport, particularly to the brain. These issues can be addressed in part, because peptide chemistry allows for a range of strategies for peptide modification or the usage of D-enantiomeric amino acid residues [89].

Eggs are not typically considered antioxidant foods, but many egg components, such as vitamins E and A, selenium, phospholipids, and carotenoids, show antioxidant properties. Moreover, egg proteins and related components (protein hydrolysates, peptides, and amino acids) exhibit multiple biological activities, including antioxidant activity [90].

Egg-derived physiologically active peptides are mainly produced from egg white proteins, but lately egg yolk has been used as a new source of functional peptides; the same goes for eggshell and egg yolk membrane. The whole egg white and yolk or even a single protein can be utilized as an initial material for the fabrication of bioactive peptides. Egg white is composed primarily of proteins (11%), being the most abundant ovalbumin (54%), followed by ovotransferrin (12%), ovomucoid (11%), lysozyme (3.5%) and ovomucin (3.5%). In addition to these, other minor proteins such as avidin, cystatin, ovomacroglobulin, ovoflavoprotein, ovoglycoprotein, and ovoinhibitors have also been recognized. The main constituent of egg yolk is lipid (31–35%), but it also contains 15–17% protein such as lipovitaline (36%), rivatin (38%), phosvitin (8%) and low-density lipoprotein. (17%) [90,91].

As chicken egg white is an exceptional source of high value proteins and bioactive peptides, one or more proteases are commonly used to produce protein hydrolysates which will then be exposed to a series of purification steps jointly with bioactivity assays to isolated powerful bioactive peptides. The main method used to attain peptides is hydrolysis with food-grade proteolytic enzymes of animal, plant, or bacterial origin. The most used proteases for obtaining protein hydrolysates are usually pepsin and alcalase for egg white in general, trypsin and papain for lysozyme and ovalbumin [90,92,93].

Some examples of antioxidant egg-derived peptides are presented here. Egg white-derived peptides, DHTKE (Asp-His-Thr-Lys-Glu), FFGFN (Phe-Phe-Glu-Phe-His), and MPDAHL (Met-Pro-Asp-Ala-His-Leu), formed via alcalase, were discovered to have antioxidant properties [92,94]. The egg white hydrolyzed by “protease P” give rise to two strongly antioxidant peptides, AEERYP (Ala-Glu-Glu-Arg-Tyr-Pro) and DEDTQAMP (Asp-Glu-Asp-Thr-Gln-Ala-Met-Pro). Pepsin hydrolyzed ovalbumin-derived peptide Tyr-Ala-Glu-Glu-Arg-Tyr-Pro-Ile-Leu has previously been reported to have angiotensin converting enzyme (ACE)-inhibitory activity and showed radical scavenging activity [92,95]. Two antioxidant tetrapeptides (Trp-Asn-Ile-Pro and Gly-Trp-Asn-Ile) were attained from the pyrolytic hydrolyzate of ovotransferrin [96]. Trp-Asn-Ile was proposed as a peptide motif involved in the significant activity of the above tetrapeptides. The ovotransferrin-derived tripeptide Ile-Arg-Trp exhibited powerful radical scavenging activity due to the tryptophan and the peptide bond between Trp and Arg [86,92]. Ovomucin-derived pentapeptide Trp-Asn-Trp-Ala-Asp has been found to decrease H_2_O_2_-induced oxidative stress in human fetal kidney cells (HEK-293) by hindering intracellular ROS accumulation. On the other side, from egg yolk, phosvitin phosphopeptides (PPP) obtained from tryptic digestion of phosvitin presented a protective effect against H_2_O_2_-induced oxidative stress in human intestinal epithelial cells [92] and, compared with intact phosvitin, PPP has shown a powerful ability to prevent lipid oxidation in the linoleic acid system and more efficient free radical capture [97].

Yu et al. chose to try to isolate some ovalbumin peptides to specifically treat AD, knowledgeable of the (among others) antioxidant properties of egg-derived peptides. They decided to take into consideration the cholinergic hypothesis and Abeta hypothesis regarding the pathomechanism of AD. The cholinergic hypothesis considers that the level of acetylcholine in the brain of AD patients is fairly low. This can happen because of the degradation produced by two cholinesterases: the first one is the true cholinesterase, AChE, and the other one is a pseudocholinesterase, BChE [98]. The AChE inhibitors are currently the most prescribed drug class for the treatment of AD [99,100]. In addition to the cholinergic hypothesis, Yu et al. considered the Abeta hypothesis (already described in paragraph 2), and they evaluated some ovalbumin-derived peptides inhibiting activity for BACE1 [98,101].

The activities of ovalbumin-derived peptides RVPSL, KLPGF, TNGIIR, and QIGLF against both AChE and BChE were evaluated by the Yu et al. Among the four peptides, KLPGF (at the concentration of 50 μg/mL) showed the greatest AChE and BChE inhibitory activity, with the inhibition values of 61.23 ± 4.73% and 3.29 ± 0.93%, respectively. Peptide TNGIIR exhibited modest AChE and BChE inhibition with the value of 58.02 ± 1.89% and 1.50 ± 0.24%, respectively. Peptides QIGLF and RVPSL had no noteworthy AChE and BChE inhibitory properties. Furthermore, the peptide KLPGF made several powerful hydrogen bonds with numerous important amino acid residues situated in the catalytic and allosteric sites of AChE a few hydrophobic interactions with AChE. The contacts between KLPGF and AChE mostly involved the resulting amino acid residues: Tyr70-Trp84-Gly118-Gly119-Trp279-Asp285-Ser286-Ile287-Phe330-Phe331-Tyr334-His440-Gly441 [99]. The peptide KLPGF significantly inhibited BACE1 activity with the IC50 value of 8.3 mmol/L. Furthermore, the peptide KLPGF produced twelve strong hydrogen bonds, two hydrophobic interactions, and three electrostatic interactions with the residues of BACE1, thus revealing its efficacy as a novel BACE1 inhibitor [101]. In another study, the tripeptide CIK was separated from ovalbumin, and was apt to efficiently hinder AChE, BChE and BACE1, with the IC50 values of 6.76, 7.72 and 34.48 μM, respectively [98].

## 5. Cholinesterase and BACE Inhibitory Activity of Egg-Derived Peptides

The cholinergic loss is one of the most prominent components of the neuropathology of Alzheimer’s disease. The cholinergic system is important for neuronal functions such as memory and learning by playing a main role in promoting neuronal plasticity. The cholinergic hypothesis considers that the level of acetylcholine in the brain of AD patients is low. This can happen because of the degradation produced by two cholinesterases: the first one is the true cholinesterase, AChE, and the other one is a pseudo-cholinesterase, BChE [98]. The hypothesis has received convincing validations, as AChE inhibitors are currently the most prescribed class of drugs for the treatment of AD [99].

The activities of ovalbumin-derived peptides RVPSL, KLPGF, TNGIIR, and QIGLF against both AChE and BChE were evaluated in the Yu et al. work. Among the four peptides, KLPGF (at the concentration of 50 μg/mL) showed the greatest AChE and BChE inhibitory activity, with inhibition values of 61.23 ± 4.73% and 3.29 ± 0.93%, respectively. Peptide TNGIIR exhibited modest AChE and BChE inhibition with the value of 58.02 ± 1.89% and 1.50 ± 0.24%, respectively. Peptides QIGLF and RVPSL had no noteworthy AChE and BChE inhibitory properties. Furthermore, the peptide KLPGF made a number of powerful hydrogen bonds with numerous important amino acid residues situated in the catalytic and allosteric sites of AChE and a number of hydrophobic interactions with AChE. The contacts between KLPGF and AChE mostly involved the resulting amino acid residues: Tyr70-Trp84-Gly118-Gly119-Trp279-Asp285-Ser286-Ile287-Phe330-Phe331-Tyr334-His440-Gly441 [99].

The peptide KLPGF significantly inhibited BACE1 activity with the IC50 value of 8.3 mmol/L. Furthermore, the peptide KLPGF produced twelve strong hydrogen bonds, two hydrophobic interactions, and three electrostatic interactions with the residues of BACE1, thus revealing its efficacy as a novel BACE1 inhibitor [101]. Furthermore, the tripeptide CIK was separated from ovalbumin, and was apt to efficiently hinder AChE, BChE and BACE1, with IC50 values of 6.76, 7.72 and 34.48 μM, respectively [98].

## 6. Beta-Sheet Breaker (BSB) Peptides as Abeta Aggregation-Inhibitor

Significant evidence indicated that the key pathological event in Alzheimer’s disease is the switch from a normal soluble Abeta into beta-sheet-rich oligomeric structures which have the capacity to form insoluble amyloid deposits with neurotoxic effects in the brain. Thus, an attractive approach against AD is the inhibition of the aggregation of Abeta through the insertion of different-sized molecules able to prevent fibril formation [102].

Thus, several studies have been based on the design of a wide range of compounds, from small peptides to large chaperones, to develop inhibitors of Abeta aggregation [103,104].

In the late 1990s, Soto and coworkers reported the results of the in vitro addition of different concentrations of a five-residue synthetic peptide, called Beta-Sheet Breaker (BSB), in the solution containing Abeta40 molecules capable of impeding their aggregation [105].

BSBs represent a class of compounds intended to bind Abeta in specific ways to inhibit and/or block its pathological conformational modification and growth. There are several causes that trigger Abeta formation, and among these are pH changes, apolipoprotein E (ApoE), especially its E4 isoform [106], α1-antichymotrypsin [106], and C1q complement factor [107], oxidative stress [108], metals [109], and proteoglycans [110].

Many distinct small compounds have been shown to avoid and/or annul Abeta polymerization in vitro, unfortunately they lack specificity, a clear mechanism of action, and sometimes show high toxicity, making them difficult to improve and to clinically use [111].

**Figure 4:**
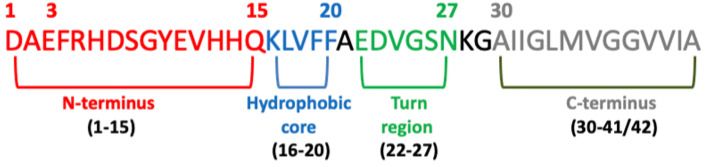
Schematic view of the Abeta domains.

Several studies have confirmed that different Abeta peptide regions contribute in a different way to aggregation and have shed light on several important interactions among specific peptide regions that control this process and are crucial for the peptide’s ability to aggregate and promote neurotoxicity. These regions are: the N-terminus (fragment 1–15) [112], the hydrophobic core (fragments 16–20) [113], the hinge or turn regions (fragments 22–27), [114] and the C-terminus (fragments 31–40/42) [115] (Figure 4).

### 6.1. N-Terminus Sequence

The neurotoxic importance of the His13–Lys16 (HHQK) at the border between the N-terminus and the hydrophobic core region in stabilizing β-sheet–rich conformation is well known. In fact, this sequence triggers the progressive steps from oligomerization, fibril propagation to plaque formation [116].

In 2020, Greco et al. synthesized and combined a natural water-soluble dipeptide, β-alanyl-L-histidine (carnosine, Car), to hyaluronic acid (HA) of two different molecular weights (HACar), showing inhibitory activity and aggregation properties against amyloidogenic species [117]. The compounds seemed to bind non-covalently to the 1–13/1–19 residues of Abeta42 in a dose dependent manner and hinder its aggregation.

In 2017, Wen used gramicidin S (GS-1), an antibacterial cyclic decapeptide, replacing the backbone ornithine residue with the amino acid arginine (GS-2). GS-2 mainly targeted the 1–12 residues (N-terminus) of Abeta42, showing inhibitory effects comparable to Ac-KLVFF-NH2 peptide against Abeta42 [118].

### 6.2. Hydrophobic Core

Bieler and Soto studied beta-sheet breakers using the Abeta self-recognition motif (17–20) to achieve specificity, stability and peptide recognition replacing proline amino acid with valine in position 18. This peptide, called iAb5p, appeared to inhibit the anomalous modification and conversion of Abeta. iAb5p further inhibited Abeta polymerization and decreased brain inflammation and neuronal loss [119]. In one rat animal model, amyloid deposition was induced by injecting non-aggregated Abeta42 in the brain. Extensive neuronal shrinkage, astrocyte and microglial activation, and amyloid plaques were present at the site of injection [120]. These signs were prevented by injection of iAb5 with Abeta42. Furthermore, iAb5, injected into the cerebral amygdala eight days after injecting Abeta at the same place, produced a significant reduction in the size of Abeta fibrils [120]. On the contrary, no effects were reported by injecting control peptides (unrelated peptides) in the same conditions [120].

In another study, the authors screened several peptides able to induce protofibril destabilization, displaying that the KKLVFFA peptide provided the maximum perturbation in the protofibril structure [121]. Jamula et al. made a step forward towards more efficient Abeta fibril dissolution by building a BSB using iAb5p as a backbone and adding three phenylalanines (iab6) to dock around position 20 in the CHC zone of Abeta, a position known to be crucial for the action of the BSB inhibitor role [122].

### 6.3. C-Terminus (31–40/42)

Regarding the Abeta C-terminus, which is rich in hydrophobic aminoacids, several peptides have been synthesized to interfere with Abeta oligomerization, [123], known as C-terminal fragments (CTFs). Li and coworkers have synthesized a series of Abeta42 CTFs, showing that, if longer than eight residues, they interrupted Abeta42 oligomerization. Among the different peptide synthetized, Abeta(31–42) and Abeta(30–42) significantly induced toxicity. [123].

Many different drug candidates with BSB function have shown great activity in vitro and in animal models, although the mechanism of action is still very blurry. What is clear is that to design a good candidate, a drug must have the ability to cross the blood-brain barrier.

## 7. The Blood–Brain Barrier (BBB) and AD

Decades of studies have established the critical role of the blood–brain barrier in AD. The blood–brain barrier (BBB) is composed of astrocytes, pericytes, and brain microvascular endothelial cells. The term blood–brain barrier describes the exclusive properties of the central nervous system microvasculature. These central nervous system vessels are non-fenestrated continuous vessels that contain some supplementary properties that allow them to tightly regulate the movement of cells, molecules and ions between the central nervous system and the blood [124]. Thus, BBB endothelial cells tightly regulate central nervous system homeostasis thanks to heavily restricting barrier capacity. This function is critical for proper neuronal function and to protect the central nervous system from injury, toxins, disease, pathogens, and inflammation [125]. The selective and restrictive proprieties of the BBB are an obstacle for drug delivery to the central nervous system. Today, the BBB is thought of as a complex and dynamic interface rather than as a static barrier [126].

Ninety-eight percent of small molecule drugs do not cross the blood–brain barrier, and one hundred percent of biologic drugs do not cross the BBB, although efforts have been made to find drug-delivery methods to bypass or modulate the blood–brain barrier. Regardless, it is essential to underline that the loss of the barrier properties is a major component of the pathology and progression in AD [127].

Although the mechanism linking Abeta accumulation and BBB dysfunction is poorly explained, it appears clear that the latter causes increased production of Abeta by activation of the β- and γ-secretase activity, establishing a vicious circle [127]. Furthermore, once that barrier is disrupted, this dysfunction leads to altered signaling homeostasis, ion dysregulation, as well as the entry of immune cells and molecules into the central nervous system. This process leads to neuronal dysfunction and degeneration. Therefore, large compounds easily invade the blood–brain barrier and normally the use of specific drug delivery systems is unnecessary.

## 8. The Insulin Effect against AD

Insulin, peptide secreted by the pancreas, plays an important role in the regulation of the glucose metabolism in the peripheral tissues. The brain was once considered an insulin-insensitive organ, but today we know that the insulin receptors are present throughout the brain and play a vital role for brain functioning [128]. Human and animal studies indicate that insulin influences cerebral bioenergetics, enhances synaptic viability and dendritic spine formation, increases the turnover of neurotransmitters, and modulates vascular function through effects on vasoreactivity, lipid metabolism, and inflammation [128]. However, some research indicates that the brain produces and uses its own insulin [129] and reaches it via the bloodstream after crossing the blood-brain barrier. The hypothesis that insulin could cross the BBB was initially suggested by Margolis and Altszuler [130], who observed a slight increase of the hormone concentration in the cerebro-spinal fluid after its peripheral infusion. Further data indicated that the insulin concentration in the brain is non-linearly correlated with the blood level of the peptide [131], implying that in the transport of insulin to the brain a saturable system is implicated [132] which appears to coincide with the insulin receptors (IR) [132]. Endothelial cell specific IR knockout (IRKO) mice intravenously injected with insulin decreased downstream insulin signaling in the hippocampus, hypothalamus, and frontal cortex [133], confirming that the kinetics of insulin signaling is controlled by the IR. Moreover, peripheral hyperinsulinemia, as it downregulates the BBB insulin receptors, could lead to lower brain insulin concentrations in patients with AD [134].

**Figure 5:**
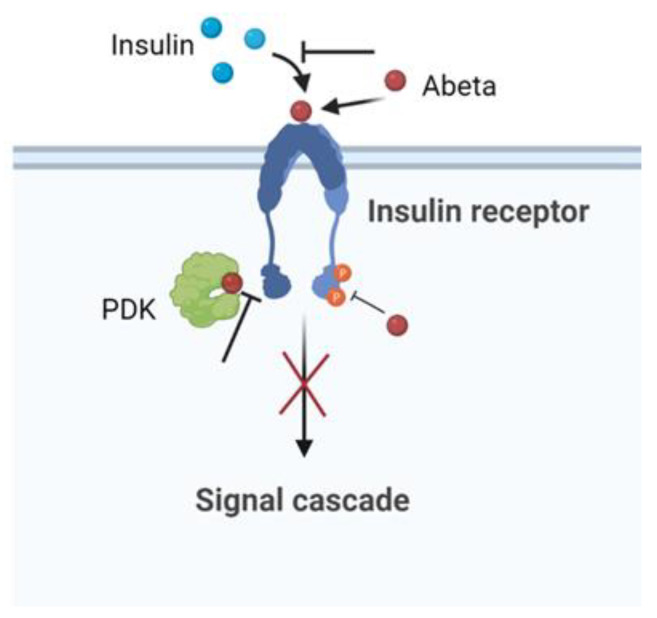
Scheme of the Abeta pathway in inducing impaired neuronal insulin signaling.

Numerous studies have suggested that insulin resistance is a key risk factor for AD [135,136,137]. Studies showed that peripheral insulin resistance in AD patients was positively correlated with Abeta deposition in the brain [138,139]. In this context, obese patients with insulin resistance have a higher risk of developing AD [1]. Reduced levels of IR and a reduced affinity of the receptor for insulin in the brain have been reported in patients with AD compared to controls [140]. Abeta induced cerebral insulin resistance with effects on insulin signaling by competing, reducing the affinity of insulin binding to its own receptor, or regulating intracellular signalling [141]. The abeta–IR interaction provoked the inhibition of the *p*-Akt insulin survival pathway. Moreover, in vitro experiments indicated that Abeta interrupted insulin signaling by blocking the association between PDK and Akt [142]. Using cultured hippocampal neurons, the amyloid derived diffusible ligands (ADDLs), that is, the soluble oligomeric forms of Abeta aggregates with the most toxic effect, were found to cause rapid redistribution of IR between the cell body and dendrites. Furthermore, the neuronal response to insulin, measured by the autophosphorylation of IR, was significantly inhibited by the presence of ADDLs. These findings suggested that insulin resistance in the AD brain is a response to Abeta, which disrupted the insulin pathway and caused a brain form of diabetes [143]. The schematic effect of Abeta in inducing impaired neuronal insulin signaling is summarized in Figure 5.

The formation of amyloid fibrils has been strongly linked to some neurodegenerative diseases such as AD, Parkinson’s disease, Creutzfeldt–Jakob disease, and motor neuron disease. It has now emerged that insulin is able to interact directly with the amyloid monomer. It has been demonstrated in vitro that the hormone was able to exert a direct effect on Abeta42 amyloid fragment aggregation. Insulin incubated with Abeta42 attenuated both Abeta42 fibril formation and its ability, in the aggregated form, to disrupt the membranes in a concentration-dependent manner [144]. Conversely, insulin deficiency may promote Abeta42 formation and toxic Tau aggregation in a mouse model of AD [145]. These data seem to show that insulin deficiency facilitated the formation of toxic Abeta42 conformers and its co-aggregation with *p*-Tau oligomers [145]. The specific interactions of insulin with Abeta are still elusive.

Recent in vitro, in vivo, and clinical studies have clearly demonstrated the important therapeutic effect played by insulin in the brain for the treatment of neurological diseases, including AD [1,9,136,146,147,148,149]. It has been demonstrated that insulin can reduce the toxicity induced by Abeta oligomers, in vitro, by the inhibition of the intrinsic apoptotic pathway [150]. Moreover, the activation of insulin signaling provides a neuroprotective mechanism to counteract oxidative stress, mitochondrial damage and neurodegeneration triggered by Abeta oligomers in neuroblastoma cells [146,147].

Intranasal administration (i.n.) of insulin in APPswe/PS1dE9 mice alleviated the cognitive deficits induced by the pathology [151]. Moreover, the insulin increased the transcription of anti-amyloidogenic proteins, such as the insulin-degrading enzyme (IDE) and α-secretase, and stimulated Abeta clearance [152]. Positive cognitive effects of brain insulin administration by the i.n. route have been demonstrated in a study in healthy humans [153]. Suzanne Craft and colleagues found that the beneficial effects of i.n. insulin on memory are not limited to healthy individuals, but can also been observed in people with MCI o early AD [154,155]. To administrate the peptide in the brain, different drug delivery systems (DDSs) have been studied to improve its targeting. Nanoparticles, liposomes, dendrimers, and nanogels have been fine-tuned with the aim of improving the drug efficacy at the target sites [149,156,157,158,159]. The i.n. route appears to be preferable for insulin brain administration as it bypasses the BBB, avoids the first pass through the liver and dilution in the systemic bloodstream, and reduces the delivery of the drug to non-targeted sites [160,161,162,163]. However, to reach significant brain levels, a dose 10 times higher than that given intravenously is required [164]. On the other hand, when intranasally administered, insulin also has to overcome some barriers, such as the nasal mucosa and the BBB, that impede absorption [165], causing its low bioavailability, which is generally less than 1% [166]. For these reasons, nasal-brain DDSs were prepared based on PVA nanogels, conjugated with insulin, obtained by electron beam irradiation and tested in cell and mouse models.

The presence of the anionic nanogel allowed the chelation of calcium and the opening of tight junctions present in the physiological barriers, protected the hormone from the attack of proteolytic enzymes of the nasal mucosa and, thanks to its high affinity for the mucous membranes, facilitated the passage of the conjugate complex with respect to the free insulin [148,149]. Further improvement of the system may lead to clinical validation of the system.

## 9. Large-Size Proteins and AD: The Case of the Heat Shock Proteins (HSPs)

HSPs are a large class of proteins well known for playing a relevant role in the protein quality control (PQC) machinery [167,168,169,170]. HSPs are normally produced under physiological conditions but become upregulated under stress conditions. Their main function is to control the correct folding of nascent proteins and prevent the aggregation of protein misfolded forms. They are also involved in cell signaling and the transport of proteins across mitochondrial membranes. HSPs have been classified in several ways based of their size, intra or extracellular localization, mechanism of action, and dependence/independence on ATP of their activity. According to their molecular weight, six families have been distinguished: small HSP (sHSP) with molecular weight lower than 40 kDa, Hsp40, Hsp60, Hsp70, Hsp90 and Hsp100. Some HSPs are very specific in their activity, others are very general, and often they may work in tandem or operate in a sequential way. Based on the mechanism of action, they have been also grouped in holding, folding, and disaggregating HSPs [171].

Holding HSPs bind partially folded proteins and maintain the substrates on their surfaces to await the availability of folding HSPs such as, for example, Hsp40 that “holds” client proteins to facilitate the folding action of Hsp70 [172]. The group of holding HSPs comprises the family of sHSPs which are ATP-independent chaperones. They are characterized by the presence of a conserved alpha-crystalline domain that recognizes hydrophobic surfaces of client partially folded proteins [173]. sHSPs are usually assembled in dynamic oligomeric structures that may easily undergo change in subunit composition for interacting with target proteins [174].

Folding HSPs (Hsp60, Hsp70 and Hsp90) are real molecular machines that rely on conformational changes induced by ATP binding and hydrolysis to mediate the refolding/unfolding of their substrates [167,168,169,170,171]. Albeit with different detailed mechanisms [175,176], folding HSPs recognize and interact with hydrophobic regions exposed by partially unfolded or misfolded proteins and use energy from ATP hydrolysis to stabilize folded conformations.

Disaggregating HSPs (e.g., Hsp104, Hsp105 and Hsp110) rely on ATP binding and hydrolysis to promote the solubilization of protein aggregates [170,171]. They belong to the AAA+ protein family (Adenosine Triphosphatases with diverse activities) as they share a common ATPase domain and structural organization in large ring-shaped complexes. They have been defined “Threading Machines” as they operate sequentially on consecutive small traits of aggregates [177].

Due to their activity in regulating the correct cellular functionality, HSPs are deemed to be powerful therapeutic agents against neurodegenerative diseases [178,179,180,181,182]. In fact, it has been proven that the overexpression of specific HSPs reduces the neurotoxicity of misfolded protein aggregates [168,171]. However, new results from in vivo and in vitro studies have demonstrated possible the negative influence of HSPs [181,183,184]. It is generally accepted that HSPs do not interact with proteins in their monomeric functional form. Rather, HSPs are capable of interfering with different steps of the aggregation process [185], working alone or in tandem or cascade. This, together with the still lacking clear recognition of which are the most dangerous species, makes a full comprehension of the various roles of HSPs extremely arduous.

The literature on the role of HSPs in AD disease is so vast that here we may only briefly recall the more recent experimental findings with reference to amyloid β-peptide and Tau proteins.

### 9.1. HSPs and Abeta

Several in vivo studies using animal or cellular models have shown that pathological dysfunctions characteristic of AD can be profitably treated with the addition of specific chaperones [179,186,187]. This is the case for the group of sHSPs that are found to be co-localized with amyloid plaques [186]. Results from several studies employing cellular and mouse models and in vitro experiments indicated that sHSPs exert a protective role and are capable of interfering either with the oligomerization of monomeric Abeta or with the elongation of fibers [186,188,189]. Recent results [184] evidenced a pathological role of a member of the Hsp40 group in promoting the aggregation of Abeta42 into small oligomers and regulating their transport to the mitochondria for degradation. This may cause AD pathology due to an excessive load of toxic species that are not efficiently degraded.

In vitro experiments on the effects of Hsp60 on Abeta40 fibrillization have shown that Hsp60 interferes with the early step of Abeta aggregation by blocking small oligomeric species that would behave as seeds for on-pathway fibrillogenesis [190]. Notably, this action does not require the presence of ATP, as would consequently be expected from the foldase activity of Hsp60. This interpretation had received further support by in vivo and ex vivo studies of the effects of Hsp60 on Abeta preformed oligomers [191].

In vivo experiments had shown that the overexpression of Hsp70 is an efficient strategy to inhibit Abeta aggregation and reduce AD symptoms [192]. In vitro experiments had shown that the addition of Hsp70 to a freshly prepared Abeta solution is capable of completely blocking the fibrillization process at sub-stoichiometric concentration [193]. The presence of co-chaperon Hsp40 and ATP strongly increases this ability, thus suggesting a mechanism of folding Abeta monomers into altered conformation not prone to aggregation. It has also proved that the addition of Hsp70 to already formed Abeta oligomers neutralized their toxicity by promoting their aggregation into larger species [179]. Similar studies for Hsp90 had shown that even this protein is capable of interfering with the early stages of the fibrillization process. However, Hsp90 activity is less enhanced by ATP presence, indicating that both folding and binding mechanisms might be operating [193].

Disaggregating chaperones constitute a further resource against the accumulation of toxic aggregates [194,195,196,197]. Both ATP-dependent HSPs (e.g., Hsp100, Hsp70) and ATP-independent sHSPs collaborate synergistically in this activity.

### 9.2. HSPs and Tau Protein

Under healthy conditions, various chaperons (sHSP Hsp40, Hsp70, and Hsp90) regulate the homeostasis of native Tau [198,199,200,201,202,203,204]. Tau is an intrinsically disordered protein capable of interacting with multiple partner molecules which could be responsible for the development of pathological forms of Tau [205,206].

Chaperones may selectively interact with different conformations of Tau by exploiting holding, folding, and disaggregating mechanisms [196]. sHSps are found co-localized with Tau plaques [187,188,189], but their mechanism of interaction with the protein remains unclear. In vitro studies on the effects of sHsp27 have shown that this protein delays the formation of Tau fibers if added at the beginning or during the lag phase [198]. Other in vitro studies have shown that sHsp22 can inhibit the aggregation of Tau and modulate its phase separation in the presence of crowding agents [199].

The group of Hsp70 is known to exert a powerful action against the formation of oligomers [198] by interacting with high affinity with hydrophobic regions of Tau. Hsp70 is also known to protect neuronal activity against the toxic effects of both oligomers and aggregates of Tau [207].

Hsp90 has been reported to bind the fibril core region of Tau protein, inducing the formation of small oligomers and inhibiting fiber formation [201,208].

Hsp104 has been reported to interact with Tau proteins by exerting a holdase effect on the monomeric form and disaggregase activity on aggregated forms [203].

Although all the papers cited so far have evinced the antagonist role of HSPs against Tau aggregation, it is worth noting that contrasting results can be found in the literature demonstrating that, in some cases, HSPs may promote Tau aggregation and accelerate the pathology [209,210,211].

## 10. Current Treatment of AD

Although scientists have made enormous progress in the pathogenesis of AD in recent years, to date the drugs available for the disease are still those produced in the 2000s. These drugs, which can temporarily mitigate cognitive and behavioral symptoms in AD, include acetylcholinesterase (AChE) inhibitors and memantine [212]. AChEs are a group of drugs that block the normal breakdown of acetylcholine (a neurotransmitter believed to be important for memory and thinking) and include Donepezil, Galantamine, and Rivastigmine. AChEs are usually prescribed to people with early-to-middle stage AD, although recent evidence recommends the use of donepezil even for people with severe AD [213]. Memantine is an N-methyl-D-aspartate receptor antagonist and is used for moderate to severe AD, and also as an add-on to AChEs. Glutamate is a neurotransmitter that, when produced in excess, causes apoptotic neuronal cell death. Memantine is believed to act by regulating NMDA receptors for glutamate, thereby reducing apoptosis.

In June 2021, the U.S. Food and Drug Administration (FDA) approved aducanumab, an intravenous anti-amyloid antibody, which is the only disease-modifying drug currently approved for AD therapy [214]. Aducanumab appears to reduce amyloid deposits in the brain, thus slowing the cognitive and functional progression of the disease. However, the European Medicines Agency did not give approval for the drug in Europe because of the lack of evidence regarding the real efficacy of the drug and some concerns about its safety [215].

Although still at the level of pre-clinical clinical trials, proteins and peptides have been explored as drugs against AD. Adults diagnosed with MCI or mild to moderate AD received 20 IU or 40 IU of insulin detemir through an intranasal delivery device. Significant effects were observed for verbal and visuospatial working memory for the subjects who received the higher dose of the protein [155,216]. Oral administration of the D-4F peptide, together with pravastin, was able to reduce the microglia and astrocyte activation and Abeta deposition and increase the cognitive function in mouse brains [217].

The development of β- and γ-secretase inhibitors failed during clinical trials because the large size of the molecules made them unable to cross the blood brain barrier [218]. 

As mentioned above, continuous brain infusion of the MT3 protein in mice reduced the oxidation levels, neuronal apoptosis, pathological hippocampal changes, and cognitive impairment occurring in AD [64].

Several other disease-modifying drugs are currently being tested in people with MCI or early AD [219], and it is hoped that soon we will have truly useful and safe drugs available to treat the disease.

## 11. Summary

In Table 1 the peptides/proteins mentioned in the paragraphs presented above, as well as their biological target or therapeutic effects, are reported.

## 12. Conclusions

Alzheimer’s disease is a progressive devastating complex pathology affecting millions of people worldwide and, thanks to the increase in life expectancy, is destined to affect an increasing number of individuals with heavy repercussions for patients, their families and for the socio-economic system that must support these events. The solution to AD remains a major medical and social challenge, and many research labs are still working hard to permanently defeat the disease by trying to identify a drug that can definitively control the cognitive impairment, functional decline and behavioral symptoms of the disease.

## Figures and Tables

**Table 1:** Peptides/proteins mentioned in the manuscript, their molecular weight, biological effect or biological target, and bibliographic references.

Peptide/Protein	MW (Da)	Biological Effect/Target
Egg-derived peptides	25–250	Antioxidant effect [89,90,97] Cholinesterase and BACE inhibitory activity [98,99,100,101]
BSB peptides	25–250	Destabilizing effect against Abeta fibrillation process [116,117,118,123] Anti-inflammatory effect [119]
DF-4 peptide	2300	Reduction of the microglia and astrocyte activation [217] Decrease of Abeta deposition [217] Increase the cognitive function [217]
Insulin	5800	Akt insulin survival pathway activation [142,146] Destabilizing effect against Abeta fibrillation process [144] Inhibition of the intrinsic apoptotic pathway and mitochondrial protection [150] Antioxidant effect [146]
MT3	6000	Maintenance of metal ion brain homeostasis [62]
ZnT3	65,000	Maintenance of Zn^2+^ brain homeostasis [66]
S100 family	10,000–12,000	Promotion of neuronal and neurite growth [68]
Copper- chelating peptides	250–1000	Ability in chelating Cu^2+^ ions, reducing ROS formation and amyloid toxicity [60,73]
HSPs	15,000–100,000	Chaperon activity [171] Reduction of the neurotoxicity of misfolded protein aggregates [168,171] Inhibition of Abeta aggregation [192] Solubilization of protein aggregates [170,171]

## Data Availability

Data reported in the present review are obtained by Scopus data base.
